# Paradoxical overexpression of MBNL2 in hepatocellular carcinoma inhibits tumor growth and invasion

**DOI:** 10.18632/oncotarget.11577

**Published:** 2016-08-24

**Authors:** Yu-Hsin Lee, Yu-Lin Jhuang, Yu-Ling Chen, Yung-Ming Jeng, Ray-Hwang Yuan

**Affiliations:** ^1^ Graduate Institute of Pathology, College of Medicine, National Taiwan University, Taipei 10051, Taiwan; ^2^ Department of Pathology, National Taiwan University Hospital and College of Medicine, National Taiwan University, Taipei 10051, Taiwan; ^3^ Departments of Surgery, National Taiwan University Hospital and College of Medicine, National Taiwan University, Taipei 10051, Taiwan; ^4^ Department of Integrated Diagnostics and Therapeutics, National Taiwan University Hospital, Taipei 10051, Taiwan

**Keywords:** hepatocellular carcinoma, alternative splicing, muscleblind proteins, hepatic progenitor cells, liver carcinogenesis

## Abstract

Pre-mRNA alternative splicing is an essential step in the process of gene expression. It provides cells with the opportunity to create various protein isoforms. Disruptions of alternative splicing are associated with various diseases, including cancer. The muscleblind-like (MBNL) protein is a splicing regulatory protein. Overexpression of MBNL proteins in embryonic stem cells promotes differentiated cell-like alternative splicing patterns. We examined the expression level of MBNL2 in 143 resected HCCs using immunohistochemistry. MBNL2 was overexpressed in 51 (35.7%) HCCs. The overexpression of MBNL2 correlated with smaller tumor size (≤ 3 cm, *P* = 0.0108) and low tumor stage (Stage I, *P* = 0.0026), indicating that MBNL2 expression was lost in the late stage of HCC development. Furthermore, patients with MBNL2-positive HCCs had a borderline better 5-year overall survival (*P* = 0.0579). In non-cancerous liver parenchyma, MBNL2 was stained on the Canals of Hering and hepatocytes newly derived from hepatic progenitor cells. The overexpression of MBNL2 in Hep-J5 cells suppressed proliferation, tumorsphere formation, migration, and *in vitro* invasion, and also reduced *in vivo* tumor growth in NOD/SCID mice. In contrast, MBNL2 depletion with RNA interference in Huh7 cells increased *in vitro* migration and invasion, but did not enhance tumor growth. These results indicate that MBNL2 is a tumor suppressor in hepatocarcinogenesis.

## INTRODUCTION

Hepatocellular carcinoma (HCC) is one of the most common types of cancer worldwide, particularly in Taiwan, Southern China, Southeast Asia, and sub-Saharan Africa. The major risk factors are hepatitis B and C infections, cirrhosis, and exposure to environmental carcinogens such as aflatoxins [1]. Molecular studies have revealed the involvement of *p53* and *β-catenin* mutations in hepatocarcinogenesis [2, 3]. However, the molecular mechanisms of HCC remain largely unclear. Most HCC cases are treated with locoregional therapy modalities such as surgical resection, transarterial chemoembolization, and radiofrequency ablation. Sorafenib is the only available effective systemic therapy, which provides an approximate 3-month survival advantage for patients in the advanced stage [4].

Most human genes are alternatively spliced [5]. Spliced isoforms often encode proteins with distinct and even antagonistic properties. Splicing variants from cancer-related genes may critically influence cancer cell biology. A cancer-related gene can express spliced isoforms that either favor or counteract the growth of cancer cells. For example, the apoptotic regulator Bcl-x has two isoforms, Bcl-xS and Bcl-xL. The Bcl-xS spliced isoform is proapoptotic, whereas the Bcl-xL spliced isoform is antiapoptotic [6]. Alternative splicing is regulated by splicing factors. Several splicing factors are overexpressed or underexpressed in cancer [7]. Mutations in splicing factor 3B subunit 1 gene (SF3B1) have frequently been identified in uveal melanoma, chronic lymphocytic leukemia, and myelodysplasia [8–10]. Alternative splicing of specific genes has been observed in HCC [11, 12]. However, the spectrum of alternative splicing and the roles of splicing factors in hepatocarcinogenesis remain unknown.

Muscleblind-like (MBNL) proteins constitute a family of RNA-binding factors that regulate developmentally programmed alternative splicing in multiple organs [13, 14]. The MBNL proteins contain two pairs of highly conserved zinc fingers, which bind to pre-mRNA to regulate alternative splicing. Mammals express three closely related MBNL genes [15]. In mice and humans, MBNL1 and MBNL2 are expressed across many tissues, including brain, heart, and muscle tissue, whereas MBNL3 is expressed primarily in the placenta [16]. The MBNL proteins are implicated in the pathogenesis of myotonic dystrophy type 1, which is a triplet-repeat expansion disease caused by CTG-repeat expansion in the 3′ untranslated region of the myotonic dystrophy protein kinase gene, leading to myotonia, muscle degeneration, reduced heart function, ocular cataracts, and nervous system dysfunction [17–19]. In myotonic dystrophy, MBNLs are sequestered away from their normal RNA targets by interacting with expanded CUG or CCUG repeats, shifting the splicing pattern toward fetal isoforms [20, 21]. Knockout of the *Mbnl1* gene leads to muscle, eye, and RNA splicing abnormalities that are characteristic of myotonic dystrophy [22]. The *Mbnl2* knockout mice develop several myotonic dystrophy-associated central nervous system features including abnormal rapid eye movement sleep propensity and deficits in spatial memory [23, 24]. MBNL1 and MBNL2 are direct, negative regulators of a large program of the cassette exon alternative splicing events that are differentially regulated between ES cells and other cell types. Knockdown of MBNL proteins in differentiated cells causes a switch to an ES-cell-like alternative splicing pattern, whereas overexpression of MBNL proteins in ES cells promotes differentiated cell-like alternative splicing patterns [25].

The relationship between stem cells and human cancer has become a critical issue in cancer research, because self-renewal is a hallmark of both cell types. Since MBNL2 regulates the splicing pattern of ES cells, it may also likely to regulate the splicing pattern of cancer cells to promote tumorigenesis [26]. The present study is aimed to study the expression of MBML2 in HCC and its role in hepatocarcinogenesis.

## RESULTS

### MBNL2 expression and distribution in HCC and non-cancerous liver

**Figure 1:**
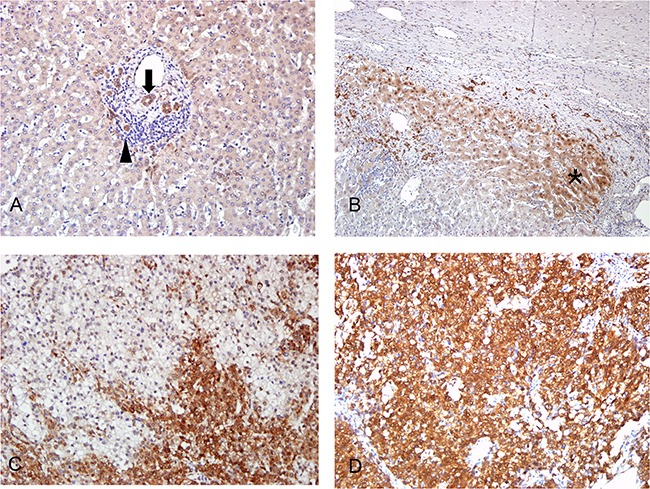
Immunostaining of MBNL2 in non-cancerous liver parenchyma and HCC **A.** In the non-cancerous part, MBNL2 was expressed in the bile ducts (arrow) and canals of Hering (arrowhead). **B.** In occasional specimens, MBNL2 was expressed in the compressed liver parenchyma (* area) adjacent to the tumor nodule. **C, D.** MBNL2 stained both cytoplasm and nuclei of HCC tumor cells. In most specimens, it demonstrated heterogeneous staining (C), but diffuse positivity was observed in some specimens (D). A, B, C, D x 200 (original magnification).

MBNL2 was detected in 84 of 143 HCC (58.7%) specimens using immunohistochemistry. The MBNL2 staining was both cytoplasmic and nuclear in the tumor cells. In the non-cancerous part, MBNL2 was expressed in the bile ducts and Canals of Hering (Figure 1A). The hepatocytes were usually not stained. However, in some specimens, MBNL2 was expressed in the compressed liver parenchyma adjacent to the tumor nodule (Figure 1B). Moreover, scattered MBNL2-positive hepatocytes were occasionally detected in the periportal liver parenchyma. The staining of tumor cells was typically stronger than that of non-cancerous tissue. The expression of MBNL2 in HCC showed a heterogeneous distribution ranging from diffuse positive (>50%) in 21 cases (14.7%), heterogeneous positive (11-50%) in 30 cases (21.0%; Figure 1C), to positive in few tumor cells (≤10%) in 33 cases (23.1%; Figure 1D).

We also analyzed the expression of MBNL1 in 31 pairs of HCC and non-cancerous liver parenchyma by real-time polymerase chain reaction (PCR). As shown in Supplementary Figure S1, the expression levels of MBNL1 were similar in HCC and non-cancerous liver parenchyma.

### Correlation of clinical and pathological factors and overexpression of MBNL2 in HCC

**Figure 2:**
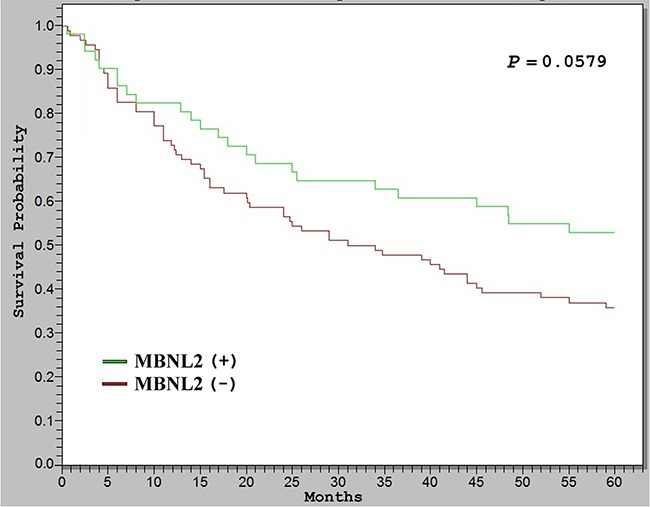
Kaplan–Meier analysis of 5-year overall survival (OS) in 143 patients with HCC The HCCs with MBNL2 protein expression correlated with borderline higher OS than the HCCs without MBNL2 protein expression (*P* = 0.0579).

To elucidate the significance of MBNL2 overexpression in HCC, we examined correlations between MBNL2 protein overexpression and major clinical and pathological features of HCC. For data presentation, HCC with more than 10% of tumor cells showing immunostaining for MBNL2 was regarded as positive. As presented in Table 1, positive MBNL2 expression in the HCCs exhibited a significant association with small tumor size (≤3 cm, *P* = 0.0108) and low tumor stage (Stage I, *P* = 0.0026). It did not correlate with other clinical or pathological parameters, such as age, gender, serum HBsAg status, serum HCV status, cirrhosis, serum α-fetoprotein (AFP) levels, tumor grade, *p53* mutation and *β*-catenin mutation. The Kaplan-Meier survival analysis showed that patients with MBNL2-positive HCCs exhibited a borderline higher 5-year overall survival rate than the patients with MBNL2-negative HCCs (*P* = 0.0579, Figure 2).

**Table 1:** Analysis of MBNL2 protein expression with various clinical and pathological features in 143 patients with surgically resectable hepatocellular carcinoma

		MBNL2 protein expression			
Variables		Total	Yes n (%)	O.R. (95% C.I.)	*P* value
Age (year)	≤ 56	64	19 (29.7)	1.0	
	> 56	79	32 (40.5)	0.62 (0.29-1.32)	0.1793
Gender	Male	108	39 (36.1)	1.0	
	Female	35	12 (34.3)	1.08 (0.45-2.61)	0.8447
HBsAg	( − )	47	16 (34.0)	1.0	
	(+)	96	35 (36.5)	0.90 (0.40-1.99)	0.7769
Anti-HCV	( − )	74	25 (33.8)	1.0	
	(+)	50	20 (40.0)	0.77 (0.34-1.72)	0.4801
Cirrhosis	( − )	85	25 (29.4)	1.0	
	( + )	58	26 (44.8)	0.75 (0.24-1.09)	0.0588
AFP (ng/ml)	≤ 200	70	26 (37.1)	1.0	
	> 200	73	25 (34.2)	1.13 (0.54-2.38)	0.7178
Tumor size (cm)	≤ 3	38	20 (52.6)	1.0	
	> 3	105	31 (29.5)	2.65 (1.16-6.10)	0.0108
Tumor grade	I-II	91	34 (37.4)	1.0	
	III-IV	52	17 (32.7)	1.23 (0.56-2.68)	0.5749
Tumor stage	I	55	28 (50.9)	1.0	
	II-III	88	23 (26.1)	2.93 (1.36-6.36)	0.0026
*p53* mutation	( − )	39	11 (28.2)	1.0	
	( + )	54	14 (25.9)	1.12 (0.40-3.12)	0.8067
β-catenin mutation	(−)	91	27 (29.7)	1.0	
	(+)	10	1 (10.0)	3.80 (0.45-83.90)	0.2763

Abbreviations: O.R., Odds ratio; C.I., Confidence interval; AFP, α-fetoprotein.

(−), designated absence; (+), designated presence.

### Expression of MBNL2 in hepatocytes newly derived from hepatic progenitor cells

**Figure 3:**
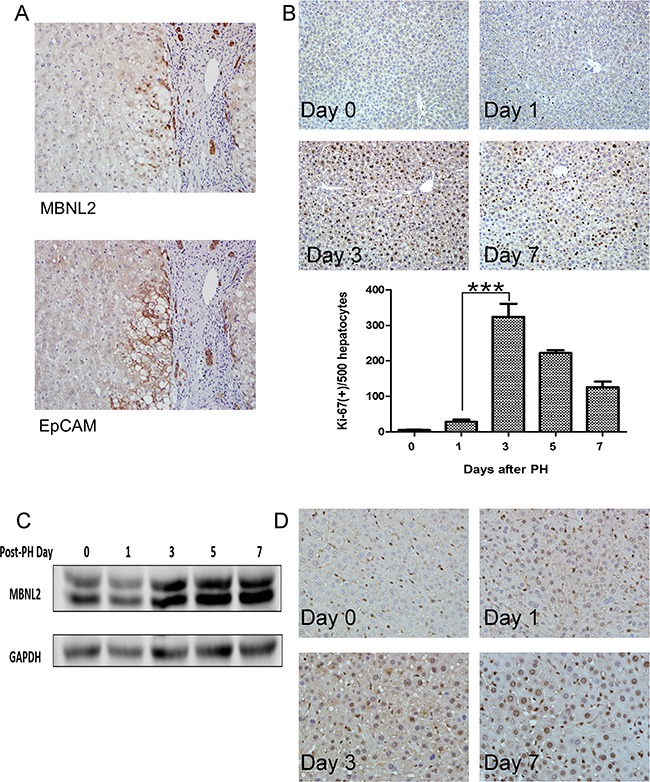
Expression of MBNL2 in hepatocytes newly derived from hepatic progenitor cells **A.** Colocalization of MBNL2 and EpCAM in the non-cancerous liver parenchyma in serial sections indicates that MBNL2 protein was induced in hepatocytes newly derived from hepatic progenitor cells. **B.** Ki-67 immunostaining confirmed regeneration of hepatocytes after two-thirds partial hepatectomy (PH) in C57BL/6 mice. The expression of Ki-67 in day 3 post-PH was significantly higher than the expression of Ki-67 in day 1 post-PH. **C.** Western blot analysis showed a gradual induction of MBNL2 protein expression up to Day 7 post-PH in C57BL/6 mice. **D.** Immunostaining also demonstrated that the MBNL2-positive cells increased gradually post-PH, and nearly all hepatocytes were positive for MBNL2 on Day 7 post-PH in C57BL/6 mice. ***P value < 0.001, Student's test.

The expression of MBNL2 was observed in certain periportal hepatocytes and compressed liver parenchyma (Figure 1B). We hypothesized that these MBNL2-positive hepatocytes were the cells newly derived from hepatic progenitor cells. Because the hepatocytes newly derived from hepatic progenitor cells expressed EpCAM [23], we stained MBNL2 and EpCAM in serial sections. As expected, we found that MBNL2 was colocalized with EpCAM in non-cancerous liver parenchyma (Figure 3A), indicating that MBNL2 was expressed in hepatocytes newly derived from hepatic progenitor cells.

### Induction of MBNL2 during liver regeneration

To elucidate whether MBNL2 is expressed during hepatocyte proliferation, we conducted two-thirds partial hepatectomy (PH) in C57BL/6 mice. In the two-thirds PH operation, the median and left lateral lobes were removed. From Day 1 post-PH, the residual liver exhibited an elevated rate of regeneration, and the growth rate reached a plateau on Day 3 post-PH, as found through Ki-67 immunostaining (Figure 3B). Then the expression of MBNL2 was evaluated at various time points using western blotting and immunostaining. The Western blot analysis indicated a gradual induction of MBNL2 expression up to Day 7 post-PH (Figure 3C). The immunohistochemical staining also showed that the MBNL2-positive cells increased gradually post-PH, and nearly all the hepatocytes were positive for MBNL2 on Day 7 post-PH (Figure 3D).

### Overexpression of MBNL2 suppressed tumor proliferation and invasion

**Figure 4:**
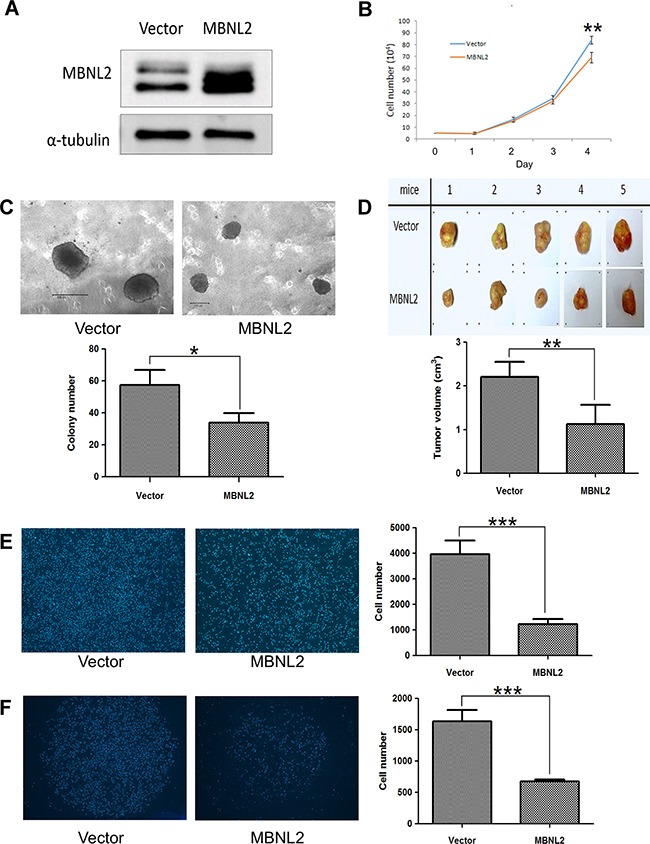
Overexpression of MBNL2 suppressed proliferation and invasion in Hep-J5 HCC cell line **A.** Western blot analysis confirmed the overexpression of MBNL2 protein in Hep-J5 cells through lentiviral transduction. **B.** Cell-counting assay indicated that overexpression of MBNL2 did not affect the anchorage-dependent cell growth. **C.** Soft agar assay demonstrated that overexpression of MBNL2 significantly suppressed the anchorage-independent cell growth. **D.** Tumorigenicity assay showed that overexpression of MBNL2 significantly suppressed the *in vivo* growth of the subcutaneous xenograft in NOD/SCID mice. **E, F.** Overexpression of MBNL2 also suppressed the migration (E) and invasion (F) ability of Hep-J5 cells significantly. (*P value < 0.05; **P value < 0.01; ***P value < 0.001, Student's test).

An analysis of MBNL2 expression in human liver cancer cell lines showed that MBNL2 was expressed at low levels in Hep-J5 cells and at high levels in Huh7 cells. Therefore, we overexpressed MBNL2 in Hep-J5 cells through lentiviral transduction. The Western blot analysis confirmed the overexpression of MBNL2 protein in MBNL2-transduced cell lines (Figure 4A). The proliferation rate was slightly reduced by MBNL2 overexpression under the anchorage-dependent condition (Figure 4B). The soft agar assay was used to assess the cell growth in the anchorage-independent manner, a characteristic of cancer cells. The results showed that both size and number of colonies decreased in Hep-J5 cells with MBNL2 overexpression compared with the vector control (Figure 4C).

The tumorigenicity assay with subcutaneous injection of Hep-J5 cells into NOD/SCID mice showed that the tumors formed by MBNL2-positice cells were significantly smaller than those formed by MBNL2-negatice cells (Figure 4D). In addition, the Boyden chamber assay showed that cell migration and invasion decreased significantly in MBNL2-positice cells (Figure 4E and 4F).

### Knockdown of MBNL2 did not affect cell growth, but enhanced tumor invasion

**Figure 5:**
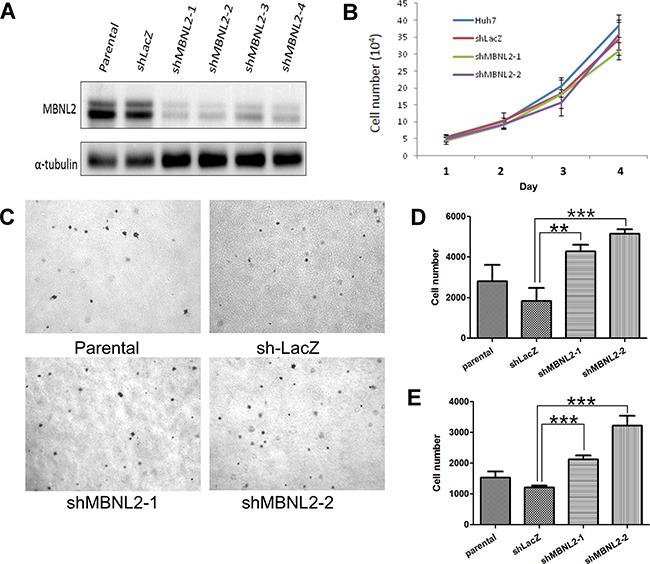
Knockdown of MBNL2 enhanced the invasion ability of HCC **A.** Western blot analysis showed that short hairpin RNAs (shRNA) #1~#4 markedly reduced the protein expression of MBNL2 in Huh7 cells. **B.** Cell-counting assay showed that the proliferation rates of parental cells, vector control, and cells with MBNL2 knockdown by shRNA #1 and #2 were similar. **C.** Soft agar assay indicated that MBNL2 knockdown did not alter the anchorage-independent growth ability in Huh7 cells. **D, E.** Boyden chamber assay showed that knockdown of MBNL2 by shRNA #1 and #2 significantly enhanced the migration (D) and invasion (E) ability in Huh7 cells. (**P value < 0.01; ***P value < 0.001, Student's test.)

To investigate the role of MBNL2 in HCC tumorigenesis of HCC, four lentiviral constructs carrying MBNL2 shRNA were used to transduce the HCC cell line Huh7. The Western blot analysis showed that all these shRNAs induced substantial reduction in MBNL2 protein expression (Figure 5A). shRNAs #1 and #2 were selected for use in subsequent studies.

We tested the effect of MBNL2 knockdown on cell proliferation. Knockdown of MBNL2 had no effect on cell proliferation under the anchorage-dependent condition (Figure 5B). The soft agar assay indicated that MBNL2 knockdown did not alter the anchorage-independent growth ability in Huh7 cells (Figure 5C). In the Boyden chamber assay, knockdown of MBNL2 markedly increased tumor cell migration and invasion (Figure 5D-5E).

### Overexpression of MBNL2 suppressed the formation of tumorspheres and expression of stem cell markers

**Figure 6:**
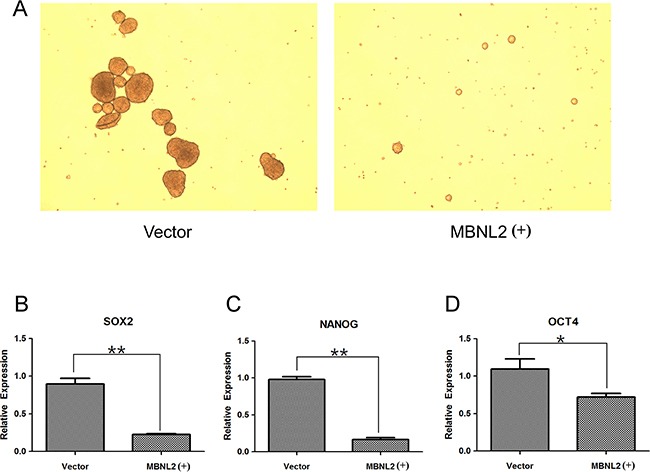
Overexpression of MBNL2 suppressed the formation of tumorspheres and expression of stem cell markers **A.** Overexpression of MBNL2 through lentiviral transduction.in Hep-J5 cells reduced the tumorsphere formation in the suspension culture. **B, C, D.** Quantitative real-time PCR assay demonstrated that overexpression of MBNL2 in Hep-J5 cells through lentiviral transduction significantly suppressed the expression of stem cell markers SOX2 (B), NANOG (C), and OCT4 (D). (*P value < 0.05; **P value < 0.01, Student's test.)

The ability to form tumorspheres is a characteristic of cancer stem cells [24]. When growing in a non-adherent plate, the formation ability of tumorspheres was reduced in MBNL2-positive Hep-J5 cells (Figure 6A). Subsequently, we evaluated the effect of MBNL2 on the expression of stem cell genes (SOX2, NANOG, and OCT4) in Hep-J5 cells. The quantitative PCR showed that the expressions of these three stem cell markers were reduced significantly in MBNL2-positive Hep-J5 cells (Figure 6B-6D).

## DISCUSSION

The role of MBNL proteins in alternative splicing in ES cells was reported by Han et al. [25]. The relationship between stem cells and human cancers is a critical issue in cancer research because self-renewal is a hallmark of both cell types. Hence, MBNL2 may be another example of cancer cells using embryonic processes to facilitate their growth and development. So we conducted this study to determine the role of MBNL2 in hepatocarcinogenesis.

In our study, MBNL2 overexpression (>10% of tumor cells) was detected in 51 of 143 (35.1%) HCCs. The finding of MBNL2 overexpression in HCC was unexpected, because the expression of MBNL2 is very low in ES cells and much higher in differentiated cells. We reasoned that the induction of differentiation in the ES cells is not altering them into terminally mature cells, but to a status highly similar to that of organ-specific stem cells. This hypothesis is consistent with our finding that MBNL2 was expressed in the Canals of Hering, where hepatic progenitor cells reside. In an adult liver, mature hepatocytes seldom proliferate and have a lifespan of more than 1 year. An adult liver has the remarkable potential to regenerate after severe parenchymal loss. When mature hepatocytes and cholangiocytes are damaged or their replication is inhibited, a reserve compartment of hepatic progenitor cells is activated. This process involves an expansion of bipotential transit amplifying progenitor cells, which can differentiate into hepatocytes and biliary cells. EpCAM marks hepatocytes newly derived from progenitor cell [27]. In our study with human tissue, MBNL2 was colocalized with EpCAM in non-cancerous liver parenchyma. We also discovered an induction of MBNL2 after PH in mice. These results indicated that MBNL2 is activated during liver regeneration and may regulate this process.

We conducted functional studies to assess the role of MBNL2 in tumorigenesis, and found that MBNL2 overexpression in Hep-J5 inhibited anchorage-dependent and -independent growth, tumorsphere formation, *in vivo* tumorigenicity, migration, and invasion. By contrast, although MBNL2 knockdown in Huh7 cells did not affect *in vitro* proliferation and anchorage-independent growth, it increased the migration and invasion ability of Huh7 cells. These findings suggest that MBNL2 is a tumor suppressor protein in HCC. MBNL2 was overexpressed in HCC; however, the rate of expression decreased in larger tumors. The expression of MBNL2 is likely to be a restraint mechanism activated in the liver regenerative process for controlling excessive hepatocyte proliferation during liver regeneration. This mechanism also functions at an early stage of hepatocarcinogenesis, but eventually, the tumor cells escape from this mechanism to form a more aggressive phenotype.

The role of MBNL1 in mediating a splicing program involved in pluripotent stem cell differentiation was reported by Venables et al. [28]. MBNL1 was downregulated in pluripotent stem cells, and MBNL-mediated splicing program was a late event in differentiation. The splicing changes occurring late during differentiation can be reversed with MBNL1 knockdown. The overexpression of MBNL2 suppressed expression levels of the stem cell markers, namely SOX2, NANOG, and OCT4, and this finding was consistent with the observations reported in previous studies. Moreover, overexpression of MBNL2 suppressed tumorsphere formation, indicating that MBNL2-overexpressing cells reduced the stemness property. Clinically, HCCs with expression of stemness-related markers are associated with increased serum α-fetoprotein levels and a poor outcome [29]. The loss of stemness property in MBNL2-overexpressed HCC cells may account for the growth and invasion inhibitory effect of MBNL2 in HCC.

Although the splicing patterns of several genes are regulated by MBNL2, identifying the chief regulating events in the tumor suppression of HCC is difficult. An appealing candidate is FXOP1. The MBNL proteins play an essential role in the negative regulation of FOXP1 exon 18b inclusion [25], which is included in ES cells and skipped in differentiated cell types. The ES cell-specific isoform of FOXP1 stimulates the expression of transcription factor genes required for pluripotency, including OCT4, NANOG, NR5A2, and GDF3, while repressing genes that are required for ES cell differentiation [30]. However, we did not observe a significant change in the ES-cell specific isoform in our HCC cell lines (data not shown). Transcriptomic analysis showed that the MBNL proteins were involved in the localization, translation, and protein secretion of several hundreds of genes [25]. The tumor suppression effect of MBNL2 is likely due to the cooperation of multiple genes regulated by MBNL2. However, further transcriptomic analysis using the HCC cell lines to clarify the key splicing events in this process is mandatory.

In conclusion, our study showed that overexpression of MBNL2 inhibits tumor cell growth and invasion, and hence a favorable prognosis for HCC patients. By contrast, knockdown of MBNL2 was associated with a more aggressive phenotype in HCC cells. Thus, MBNL2 might serve as a new potential target of gene therapy for HCC.

## MATERIALS AND METHODS

### Patients and samples

A total of 143 unifocal primary HCCs surgically resected from patients at National Taiwan University Hospital (NTUH) from July 1981 to September 2001 were used on this study retrospectively. All resected tumors underwent detailed pathological assessment, and all patients received regular follow-up in NTUH. This study was approved by the Ethics Committee of NTUH (Approval No. 201411025RINB), and all study procedures were conducted in accordance with ethical guidelines. All study participants provided written informed consent, which was approved by the Ethics Committee of NTUH. The anonymity of all patients was maintained, and all specimens were blindly analyzed. The HCC patients included 108 males and 35 females, with a mean age of 55.6 years. Serum hepatitis B surface antigen (HBsAg) was positive in 96 (67.1%) patients, and hepatitis C antibody was positive in 50 (40.3%) patients, 18 of which were positive for both. All patients exhibited adequate liver function reserve at the time that they received curative liver resection. No patients had distant metastasis. They also had not received anticancer treatments before undergoing surgery, such as transcatheter arterial chemoembolization, percutaneous ethanol injection therapy, radiofrequency ablation, or chemotherapy.

### Immunohistochemical analysis of MBNL2 expression

MBNL2 was detected in the formalin-fixed paraffin-embedded tissue sections of HCC and liver tissue using a labeled streptavidin-biotin method. The tissue sections were deparaffinized and rehydrated. They were later immersed in a 10 mM citrate buffer (pH = 6.0) and incubated in a microwave oven at 100°C for 10 min. Endogenous peroxidase activity was blocked by incubating the sections in 0.5% hydrogen peroxide for 10 min at room temperature. After blocking with 5% fetal bovine serum (FBS), the sections were incubated with primary antibodies at 4°C overnight. Next, the slides were incubated with a polymer-HRP reagent (BioGenex, San Ramon, CA, USA). Peroxidase activity was visualized using a diamino-benzidine tetrahydroxychloride solution (BioGenex). The sections were counterstained with hematoxylin. For negative controls, the primary antibody was replaced with 5% FBS. The primary antibodies used were mouse monoclonal Ab against human MBNL2 (sc-136167, 1:100, Santa Cruz Biotechnology, Santa Cruz, CA, USA), rabbit monoclonal Ab against human EpCAM (N3C3, 1:100, GeneTex, Irvine, CA, USA), and rabbit anti-Ki67 (SP6, 1:150, NeoMarker, CA, USA). One pathologist who was blinded to the patients' outcomes calculated the percentage of positive cells based on 5 independent microscopic fields (× 400 magnifications) for each slide to ensure the representativeness and homogeneity of all specimens. All tumor cells within each microscopic field were counted, and the positive rate of MBNL2 was calculated. For data presentation, the specimens positive for MBNL2 immunostaining in a tumor were categorized as diffuse expression (>50%), heterogeneous MBNL2 expression (11-50%), or MBNL2 expression in few tumor cells (≤10%).

### Follow-up observation examination

All patients had been followed up for more than 5 years or until death, whichever occurred earlier. Among the 227 study patients, 60 (42%) survived longer than 5 years. Following surgery, all patients received laboratory examinations, including assessment of serum AFP level, at 1-to 6-month intervals, and liver ultrasonography of the liver at 3- to 12-month intervals. Computed tomography and/or magnetic resonance imaging were used to confirm and differentiate intrahepatic recurrence and/or distal metastasis in the patients with clinical signs of recurrence. Depending on the tumor site, the tumor size, the number of tumors, the level of liver function, and the patient's condition, tumor recurrence was treated by a second resection, percutaneous ethanol injection, transarterial chemoembolization, radiofrequency ablation, or chemotherapy. All patients had an equal opportunity to access all the therapeutic modalities supported by the Bureau of National Health Insurance, Taiwan

### Partial hepatectomy model for liver regeneration

C57BL/6 mice were purchased from the National Laboratory Animal Center, Taiwan. Six-week-old C57BL/6 mice were used to evaluate the role of MBNL2 in liver regeneration. Before the surgical procedure, the mice were anesthetized with an intraperitoneal injection of Tribromoethanol (Avertin^®^) (0.2 mL/10 g of body weight). Partial hepatectomy (PH) was performed by removing the left and median lobes of the liver. Approximately 70% of the liver was resected after ligation with 3–0 silk sutures around the base of each lobe [31]. The mice were sacrificed on postoperative days 0, 1, 3, 5, and 7. Parts of the remaining liver tissues were fixed in 10% formalin and embedded in paraffin for histological examination, and parts were cryopreserved for RNA and protein extraction. Euthanasia was performed by carbon dioxide inhalation at the time when the animal was sacrificed and all efforts were performed to minimize the suffering. This study was performed in compliance with the strict rules of the Institutional Animal Care and Use Committee (IACUC) of the National Taiwan University College of Medicine and College of Public Health (IACUC Approval No. 20130279).

### Cell culture

Two HCC cell lines (Hep-J5 and Huh7) and the virus-package cell line Human Embryonic Kidney (HEK) 293T cells were maintained in a Dulbecco modified Eagle's medium (DMEM) supplemented with 10% FBS, 100 U/mL of penicillin, 100 μg/mL of streptomycin, nonessential amino acids, and 1 mM sodium pyruvate, before they were incubated at 37°C in a water-saturated atmosphere of 5% CO_2_/95% air. Cell line authentication was achieved by genetic profiling using polymorphic short tandem repeat loci (Promega, Fitchburg, WI, USA).

### Plasmid, transfection, and lentiviral infection

The open reading frame of *MBNL2* was purchased from Origene (Rockville, MD, USA) and subcloned to the lentiviral vector pCDH-CMV-MCS-EF1-Puro (System Biosciences, Palo Alto, CA, USA), and were subsequently cotransfected with 2nd Generation Packaging System Mix (Applied Biological Materials, Richmond, BC, Canada) into the lentiviral package cells HEK 293T to produce lentiviral particles using the TurboFect reagent (Fermentas, Glen Burnie, MD, USA). After incubating with medium containing lentiviral particles for 2 days, the target cells were treated with puromycin (2 μg/ml, Clontech) for 2 weeks to select cells with stable integration of lentiviral vectors.

### RNA interference

The clones of short hairpin RNA (shRNA) in lentiviral vectors were constructed by the RNAi Consortium [32], and were distributed by the RNAi Core Laboratory of Academia Sinica. The selected target sequences were shMBNL2-1 (5′- CGGCTATTAGCTTTGCTCCTT -3′), shMBNL2-2 (5′- GCTAGTGCTGCTATCTCATAT -3′), shMBNL2-3 (5′- CAACACCGTAACCGTTTGTAT -3′) and shMBNL2-4 (5′- CCAGCAGATGCAATTTATGTT -3′). For lentivirus production, HEK 293T cells were transfected with 4 μg of pLKO.1 lentiviral vector, with 0.4 μg of envelope plasmid pMD.G, and 3.6 μg of packaging plasmid pCMVΔR8.91. Viruses were collected 24 h and 48 h post transfection. The HCC cells were infected with the lentiviruses for 16 h. A fresh medium containing 2 μg/mL of puromycin was added for 7 days for drug-resistant cell selection.

### Soft agar (anchorage-independent growth) assay

In total, 5 × 10^3^ cells were trypsinized and seeded into 0.35% top agar with DMEM and spread onto 6-well plates containing 0.5% bottom agar with DMEM. The cells were grown at 37°C for 14 days and colonies were counted after staining with 0.05% P-iodonitrotetrazolium violet dye overnight. Colonies larger than 100 μm were counted. Experiments were done in triplicate and repeated twice.

### *In vitro* tumorsphere formation

For culture of tumorspheres, 2 × 10^6^ cells were seeded in a 58-mm nonadhesive petri dish and maintained in DMEM supplemented with 10% FBS, sodium pyruvate, nonessential amino acids, and penicillin/streptomycin. Cells were incubated at 37°C in 5% CO_2_. The medium was changed 3 times per week. When the medium was changed, the supernatant-containing cells were collected gently into a 15-mL tube. It was centrifuged for 3 min at 80 × *g*. The medium was aspirated carefully from the tube without damaging the cell pellet. The cells were resuspended in 700 μL of a fresh culture medium and dissociated by pipetting up and down 5–10 times using a pipette with a 200-μL tip. The cells were later transferred onto a Petri dish.

### Western blot analysis

Protein samples (50 μg each) were separated through 10% sodium dodecyl sulfate–polyacrylamide gel electrophoresis (SDS-PAGE), and subsequently electrotransferred onto nitrocellulose membranes (Amersham, Buckinghamshire, UK). The membranes were allowed to react with the primary and secondary antibodies at optimal dilution, and the immunoreactive signals were detected using an enhanced chemiluminescence kit (Millipore, Bedford, MA). The primary antibodies used were mouse monoclonal Ab against human MBNL2 (sc-136167, 1:500, Santa Cruz Biotechnology, Santa Cruz, CA, USA), Glyceraldehyde-3-phosphate dehydrogenase (GAPDH) (GTX100118, 1:4000, GeneTex, Irvine, CA, USA), and α-tubulin (GTX102078, 1:10000, GeneTex, Irvine, CA, USA). The immunoreactive signals were detected using an enhanced chemiluminescence kit (Millipore, Bedford, MA, USA).

### Real-time PCR analysis

Total RNA was isolated from the tissue specimens by using the Trizol reagent (Life Technologies, Invitrogen, Carlsbad, CA, USA), according to the manufacturer's instructions. 2μg of RNA was reverse-transcribed using oligo (dT)_18_ primer and the cDNA Synthesis Kit #K1632 (Thermo Fisher Scientific, Marietta, OH, USA). The SYBR green-based real-time PCR was performed to determine the levels of target genes by using the ABI PRISM 7900HT Sequence Detection System (Applied Biosystems, Foster City, CA, USA). GAPDH, a housekeeping gene, served as a control for RNA quantity. The primers for Sox2 were Sox2-F (5′-ACACCAATCCCATCCACACT-3′) and Sox2-R (5′-GCAAACTTCCTGCAAAGCTC-3′). The primers for Nanog were Nanog-F (5′-GATT TGTGGGCCTGAAGAAA-3′) and Nanog-R (5′-AAGT GGGTTGTTTGCCTTTG-3). The primers for Oct4 were Oct4-F (5′-GAAGGATGTGGTCCGAGTGT-3′) and Oct4-R (5′-GTGAAGTGAGGGCTCCCATA-3′). The primers for MBNL1 were MBNL1-F (5′-CTGCCGAACATCTGACTAGC-3′) and MBNL1-R (5′- TCGTCCTTTACTCTAACCAAGCA-3′). The primers for GAPDH were GAPDH-F (5′-AGCCTCAAGATCATCAGCAATGCC-3′) and GAPDH-R (5′-TGTGGTCATGAGTCCTTCCACGAT-3′). In a volume of 20 μL of a PCR reaction, 1 μL of the complementary DNA template was mixed with 10 μL of 2× Power SYBR^®^ PCR master mix (Applied Biosystems), 200 nM of paired primers, and distilled water. PCR amplification included initial incubation at 50 °C for 2 min, denaturing at 95 °C for 10 min, and 40 cycles of denaturing at 95 °C for 15 s and annealing at 60 °C for 1 min. Melting curves were analyzed after each run to verify the size of the PCR product.

### Boyden chamber invasion assay

Modified Boyden chambers with filter inserts (pore size, 8 μm) coated with Matrigel (40 μg, Collaborative Biomedical, Becton Dickinson Labware, San Jose, CA, USA) in 24-well dishes (Nucleopore, Pleasanton, CA, USA) were used for the invasion assays. Cells (2 × 10^4^) in 100 μL of serum-free DMEM were placed in the upper chamber, and 550 μL of DMEM with 10% FBS were placed in the lower chamber. After 24 h or 48 h in culture, the cells were fixed in 4% paraformaldehyde in PBS for 15 min and then stained with 0.3 mM DAPI (4,6-diamidino-2-phenylindole) in 0.1% PBST (phosphate buffered saline with Tween 20) for 15 min at room temperature. Cells on the upper side of the filters were removed with cotton-tipped swabs, and the filters were washed with PBS. Cells on the underside of the filters were viewed and counted under a fluorescent microscope (Olympus IX70) coupled with a digital camera. Each group was plated in triplicate in each experiment, and each experiment was performed three times. The cell motility assay was done in the same way as invasion assay except the filters were not coated with Matrigel.

### Tumorigenicity assay

NOD/SCID mice (female, 4-6 weeks of age) were procured from the National Taiwan University Laboratory Animal Centre and accommodated for 7 days for environmental adjustment prior to experimentation. Cells were trypsinized, resuspended in serum-free DMEM, and injected subcutaneously (1 × 10^6^ cells in a total volume of 0.1 mL) into both flanks. The mice were observed weekly for tumor development over 5–8 weeks. The final tumor weights were recorded at the time of sacrifice.

### Statistical analysis

The data analyses were performed using the Epi Info computer software, version 7.1.0.6 (Centers for Disease Control and Prevention, Atlanta, GA, USA). A univariate analysis was used to examine whether the immunohistochemical markers correlated with the clinical and pathological parameters using the χ2 and Fisher's exact tests. The cumulative overall survival rates after tumor resection were calculated using the Kaplan-Meier method, and the differences in the survival curves were analyzed using the log rank test. A two-tailed *P* value of less than 0.05 was considered to indicate a statistically significant relationship.

## SUPPLEMENTARY FIGURE


