# Supplementary material for: Paradoxical overexpression of MBNL2 in hepatocellular carcinoma inhibits tumor growth and invasion
Source: Oncotarget. 2016 Aug 24;7(40):65589–601. doi: 10.18632/oncotarget.11577 (PMC5323177; doi:10.18632/oncotarget.11577)
Supplement: Supplementary file 1 [file oncotarget-07-65589-s001.pdf]

Paradoxical overexpression of MBNL2 in hepatocellular carcinoma inhibits tumor growth and invasion

SUPPLEMENTARY FIGURE

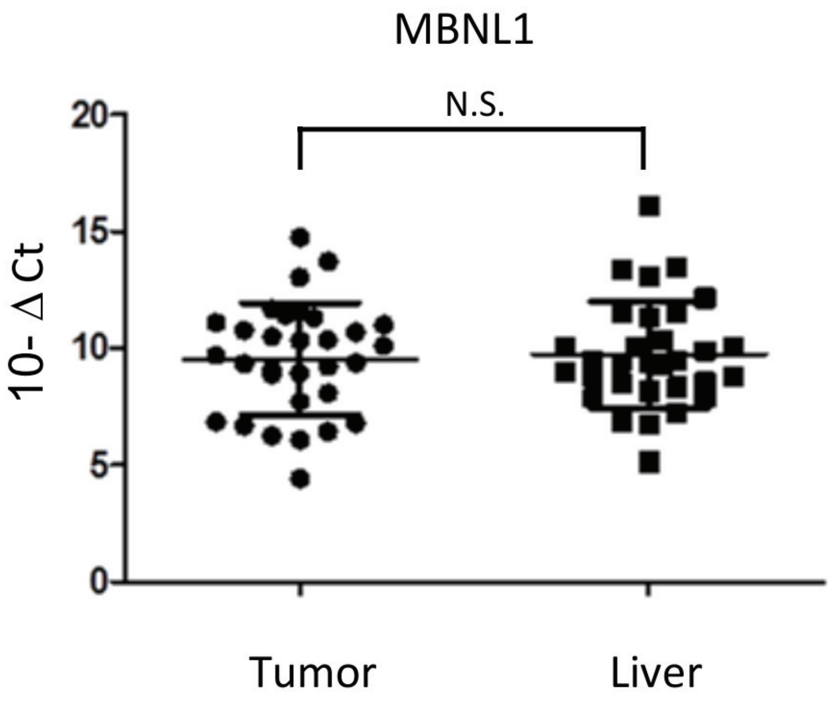

Supplementary Figure S1: Real-time PCR assay showed the expression levels of MBNL1 were similar in HCC and non-cancerous liver parenchyma.
